# Post-testicular sperm maturation in ancient holostean species

**DOI:** 10.1038/s41598-023-46900-8

**Published:** 2023-11-13

**Authors:** Viktoriya Dzyuba, William L. Shelton, Ana E. Hiott, Jacky Cosson, Olga Bondarenko, Vitaliy Kholodnyy, Borys Dzyuba

**Affiliations:** Faculty of Fisheries and Protection of Waters, South Bohemian Research Center of Aquaculture and Biodiversity of Hydrocenoses, University of South Bohemia in Ceske Budejovice, Zátiší 728/II, 389 25 Vodňany, Czech Republic

**Keywords:** Physiology, Zoology

## Abstract

Fish speciation was accompanied by changes in the urogenital system anatomy. In evolutionarily modern Teleostei, male reproductive tracts are fully separated from the excretory system, while in evolutionarily ancient Chondrostei and Holostei, the excretory and reproductive tracts are not separated. Sturgeon post-testicular sperm maturation (PTSM) occurring as a result of sperm/urine mixing is phenomenologically well described, while, in holosteans, functional intimacy of seminal ducts with kidney ducts and the existence of PTSM still need to be addressed. In *Lepisosteus platostomus* (Holostei), sperm samples were collected from testes (TS), efferent ducts (EDS), and Wolffian ducts (WDS). While WDS was motile, no motility was found in TS and EDS. The existence of PTSM was checked by in vitro PTSM procedure. After TS and EDS incubation in seminal fluid from WDS, no more than 5% motile spermatozoa were observed in TS, whereas in EDS the motility percentage was up to 75%. Experimental dyeing of urogenital ducts in gars and sturgeons revealed some differences in the interconnection between sperm ducts and kidneys. It is concluded that post-testicular sperm maturation occurs in gars and suggests that infraclass Holostei occupies an intermediate evolutionary position between Teleostei and Chondrostei in the anatomical arrangement of the urogenital system.

## Introduction

The arrangement of the male genital tract in higher vertebrates (sequence of testes, epididymides, and seminal vesicles, which are additionally interrelated with several other glands) is an arena for complicated networks of spermatozoon-specific cellular processes related to post-testicular sperm maturation (PTSM)^1^. Even though the excretory (urinary) and reproductive (genital) systems in higher vertebrates are often treated as a single system, the urogenital system, they are physically and functionally separated. The anatomical separation of these two systems has excluded the physiological role of excretory products in gamete physiological processes.

In contrast to higher vertebrates, actinopterygian fishes represent the group of animals with diversified means of external fertilization, which seems to be linked with the simplification of male reproductive system structure as extant taxa of these animals possess neither epididymides nor seminal vesicles. Moreover, actinopterygian fishes have groups with different levels of anatomical association of urinary and genital systems^2^. Actinopterygii can be subdivided into the group with fully separated urinary and reproductive tracts (Teleostei), the group with common urogenital ducts (Chondrostei), and the group with partially connected ducts (Cladistia, Holostei). Notably, the resemblance of urogenital system anatomy in holostean and chondrostean fishes to that of certain amphibians rather than other fish groups underscores the diversity of evolutionary pathways of urogenital structure anatomy in vertebrates.

It was shown that the anatomical association of urinary and genital systems in some fishes (namely sturgeons) could be closely related to PTSM^3,4^. More precisely, males of Chondrostei (sturgeons and paddlefishes) possess a so-called “primitive” excretory system in which sperm ducts enter the kidneys, where spermatozoa mix with urine before being released into the environment during spawning. Sturgeon testicular spermatozoa were found to become motility-competent only after their transit through the kidneys or after in vitro maturation (incubation in the urine or seminal fluid from already mature sperm collected from the Wolffian ducts)^3^. In this way, a complicated sequence of molecular events in the epididymis is replaced by processes in the Wolffian duct (the only post-testicular organ), where sperm maturation occurs after sperm mixing with kidney products. It has been further elucidated that changes in (1) ionic environment, (2) sensitivity of spermatozoa to calcium ions (Ca^2+^), (3) antioxidant enzymes and proteolytic activities, and (4) content of macroergic phosphates are involved in this maturation process^3,5–8^.

**Figure 1:**
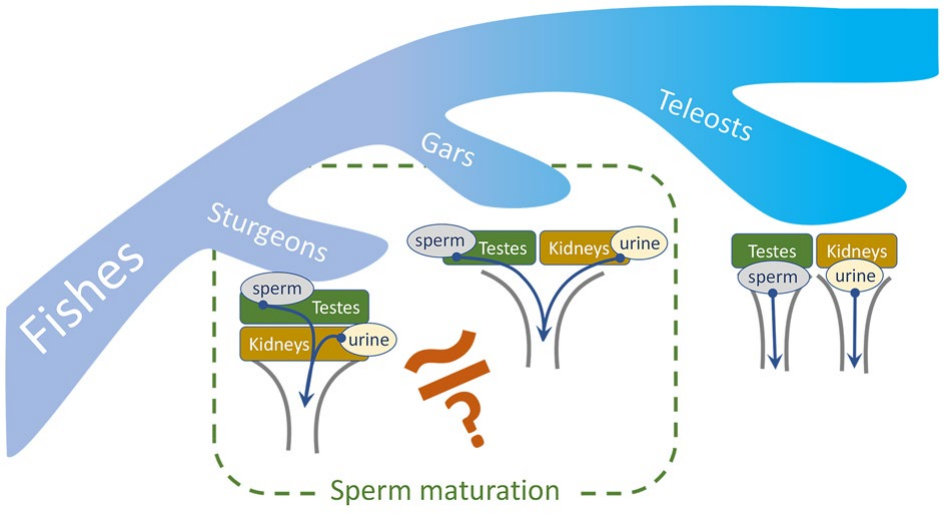
Schematic representation of actinopterygian fish groups differing in anatomical association of urinary and reproductive systems. In dashed rectangle, fish groups in which sperm maturation process is known or supposed to occur.

Unlike sturgeons, the sperm biology of other evolutionarily ancient fishes, meaning holostean fishes (presented by only eight extant species), is poorly known. In Holostei, in contrast to the close connection between the testis and kidney in sturgeons and complete separation of sperm and urinary ducts in teleosts, some intermediate situation is believed to occur: sperm could be potentially mixed with urine, but direct contact of seminal ducts with kidney is still required to be confirmed (Fig. 1). For the moment, not much is known about the exact positional relationship between kidneys, testes, and the corresponding ducts. To top it off, this scanty information is not easy to generalize, as quite different notions can be found^9–13^. Moreover, despite multi-faceted interest in studies of Holostei (general characteristics of reproductive biology^14,15^, histology of reproductive organs^16^, testis development^17^, spermatozoon ultrastructure^18^, embryo, larva, and juvenile development^17,19–22^, genome studies for clarification of vertebrate evolution^23–25^, life history and status of different gar species with recommendations for management^26–29^), we found no information on gar sperm motility, nor possible effects of kidney products on spermatozoa. This is quite surprising, considering a unique structure of their reproductive system.

We hypothesize that the specific physiology of spermatozoa in ancient fish species is explained by the physical connection between the testis and kidney, which determines the physiological role of excretory products in a complicated network of cellular processes of sperm maturation. Evolutionarily close to sturgeons holostean fishes can represent the point of divergence leading to and culminating in the complete separation of reproductive pathways and the excretory system in modern teleostean fishes. This intermediate position of holostean fishes will be manifested in the specificity of sperm maturation process. Moreover, studying this process in gars, representatives of Holostei, will contribute to understanding the evolution of urogenital system anatomy and sperm maturation strategies in vertebrates, not only in fishes.

## Results

### General characteristics of sperm samples

**Figure 2:**
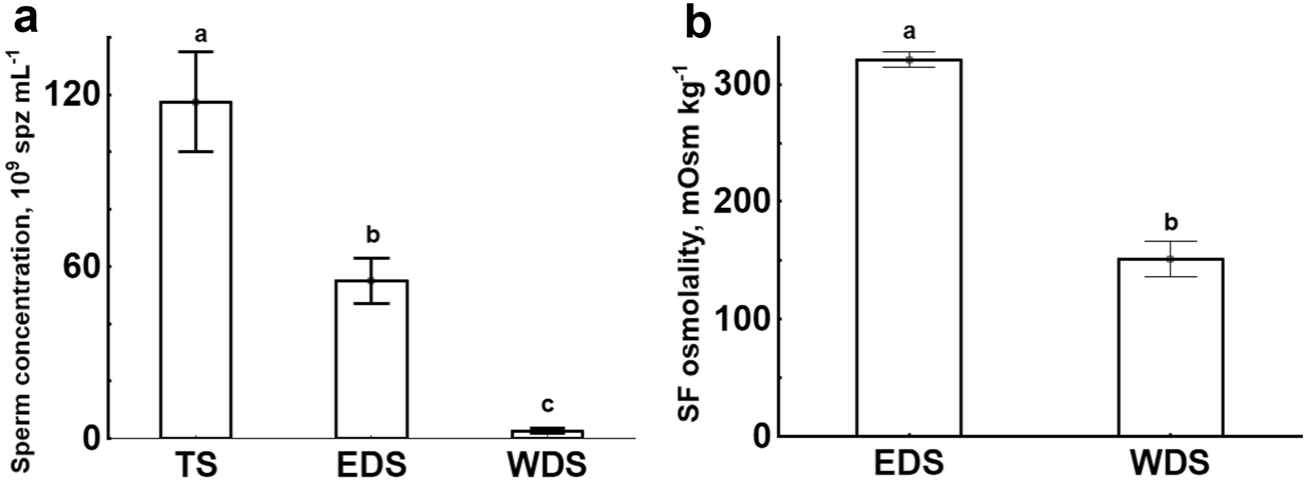
Sperm concentration and seminal fluid osmolality in different types of gar sperm samples. (**a**)—Sperm concentration, × 10^9^ spz mL^−1^. (**b**)—seminal fluid osmolality, mOsm kg^−1^. TS—sperm collected from testes, EDS—sperm collected from efferent ducts, WDS—sperm collected from Wolffian ducts. Values with different letters are significantly different (One-way ANOVA, Tukey test, *P* < 0.05).

Three types of sperm samples were collected: sperm from the testes (TS), from efferent ducts (EDS), and from Wolffian ducts (WDS). They were shown to differ significantly in sperm concentration (TS: 117.4 ± 52.1 × 10^9^ spz mL^−1^; EDS: 55.0 ± 24.1 × 10^9^ spz mL^−1^; WDS: 2.6 ± 1.9 × 10^9^ spz mL^−1^) (Fig. 2a). Seminal fluid (SF) osmolality was also significantly different: SF osmolality of EDS was 321 ± 17 mOsm kg^−1^, while in samples of WDS it was almost two times lower (151 ± 33 mOsm kg^−1^) (Fig. 2b). Osmolality of SF from TS was not measured as those samples were of low volume and extremely viscous that complicated SF obtaining.

### Motility and sperm kinematic parameters of sperm samples before and after in vitro maturation

**Figure 3:**
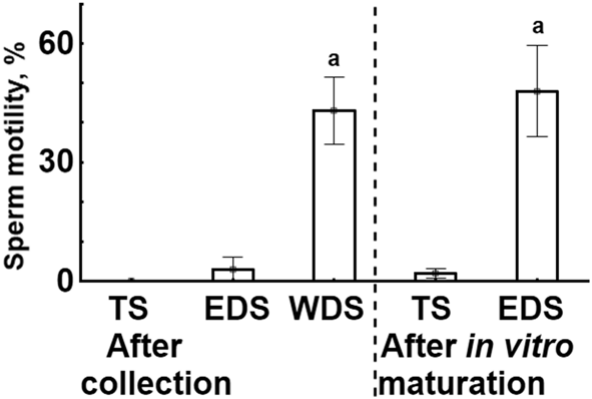
Sperm motility percent (%) in different types of gar sperm samples before and after in vitro maturation. TS—sperm collected from testes, EDS—sperm collected from efferent ducts, WDS—sperm collected from Wolffian ducts. Values marked with a small letter a are not different (Student t-test, *P* = 0.74).

Spermatozoa from Wolffian ducts were motile in all studied samples (43 ± 19%), while no motility was found in samples of TS, and only in one sample motility of 15% was found in EDS (Fig. 3). After 40 min incubation of TS and EDS in SF obtained from WDS (in vitro maturation), in two TS samples motility of 5% was observed, whereas, in contrast, in EDS samples the motility was observed in all samples (48 ± 26%). That is why only samples of WDS and EDS were subjected to statistical analysis, and no significant difference between the groups was found.

The first approach for the analysis of sperm kinematic parameters was performed by multivariate exploratory techniques using principal component (PC) analysis. Altogether, 5462 spermatozoa were analyzed in the study. Results of the PC analysis are presented in Table 1, indicating that PC1 was highly and positively correlated with all kinematic parameters except amplitude of lateral movement of the sperm head (ALH). At the same time, PC2 was positively correlated with curvilinear velocity (VCL) and ALH and highly negatively correlated with linearity of the path (LIN), straight-line velocity (STR), and oscillation of the track (WOB). The PC3 was highly associated only with beat-cross frequency (BCF) (see principal components loadings). Eigenvalues for PC1–PC3 were in the range of 3.74–0.76. Percent of explained variances was calculated to be 47, 32, and 10% for PC1, PC2, and PC3, respectively, corresponding to an 89% cumulative proportion of variance explained.

**Table 1:** Results of principal component analysis of the entire dataset of sperm motility parameters.

		Principal component		
		PC1	PC2	PC3
Principal component correlations (loadings)	VCL	0.609483	0.777536	− 0.007012
	VSL	0.935854	0.191805	− 0.097653
	VAP	0.840206	0.472066	− 0.159249
	LIN	0.767930	− 0.601940	− 0.088188
	STR	0.558662	− 0.624548	0.085516
	WOB	0.749973	− 0.395169	− 0.270586
	BCF	0.554944	− 0.102565	0.795778
	ALH	0.128492	0.890426	0.083527
Eigenvalue		3.74	2.58	0.76
Proportion of variance explained		46.8	32.2	9.5
Cumulative proportion of variance explained		46.8	79.0	88.5

**Figure 4:**
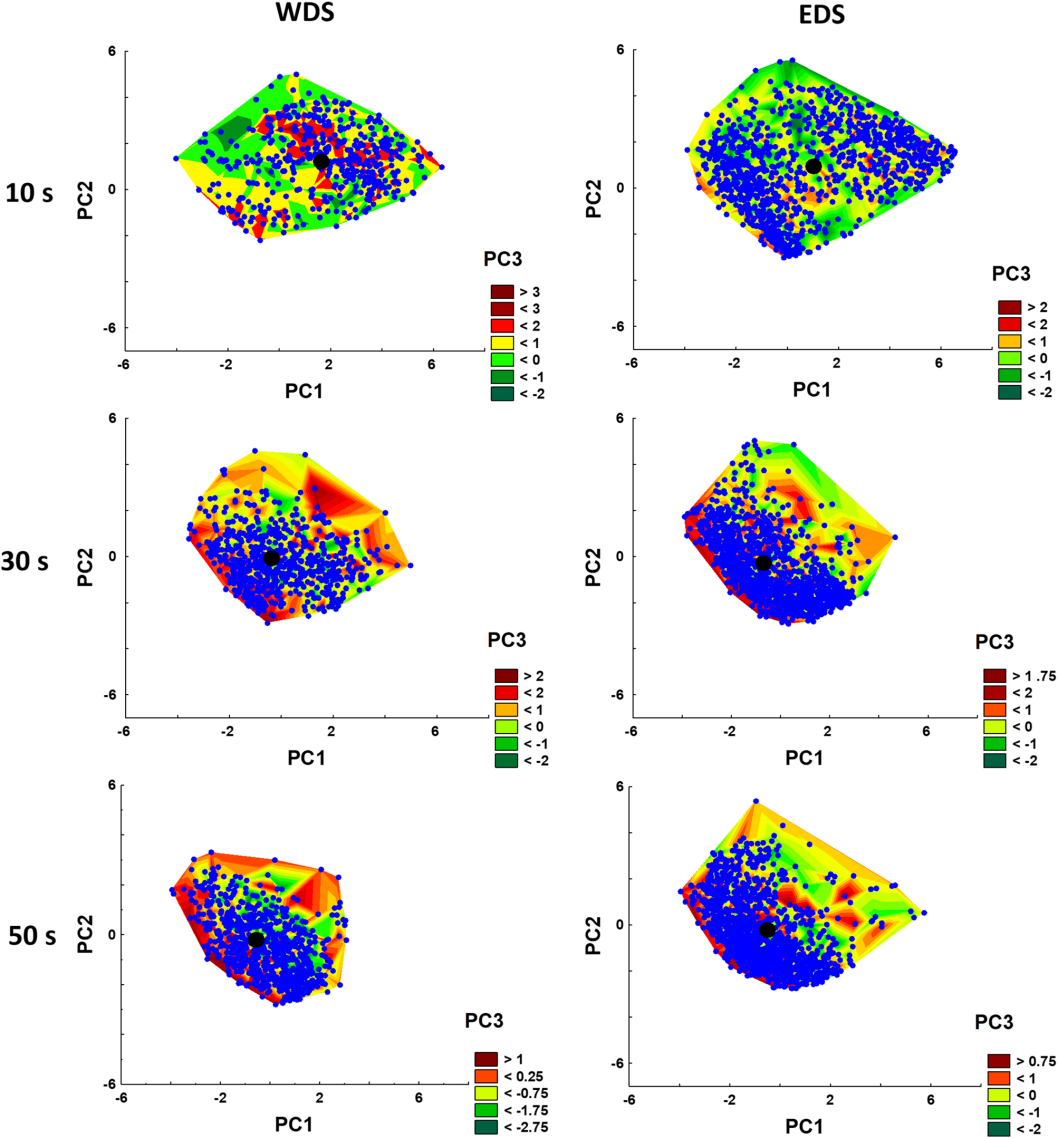
Wafer plots of three principal components (PC1, PC2, PC3), obtained after sperm kinematic parameters analysis in samples of Wolffian duct sperm (WDS) and efferent duct sperm (EDS) after in vitro maturation at different post-activation times. Dots represent individual spermatozoon in PC1–PC2 coordinates. Black dots are centroids of corresponding datasets (precise coordinates are presented in Table 2).

The obtained data on PC1–PC3 coordinates for each spermatozoon are presented as Wafer plots at 10, 30, and 50 s post activation of Wolffian duct sperm and efferent duct sperm after in vitro maturation in Fig. 4. Generally, the most remarkable changes in motility parameters were observed at 30 s post-activation time, at which centroids of corresponding datasets were characterized by a shift of PC coordinates from positive to negative values (see Fig. 4, Table 2).

**Table 2:** Principal component coordinates of centroids of datasets presented in Wafer plots (Fig. 4).

Sperm type	PC number	Post-activation time, s		
		10	30	50
WDS	1	1.67	− 0.09	− 0.22
	2	1.29	− 0.22	− 0.38
	3	0.47	− 0.06	− 0.55
EDS	1	0.77	− 0.38	− 0.68
	2	0.79	− 0.48	− 0.35
	3	0.29	0.01	− 0.06

**Figure 5:**
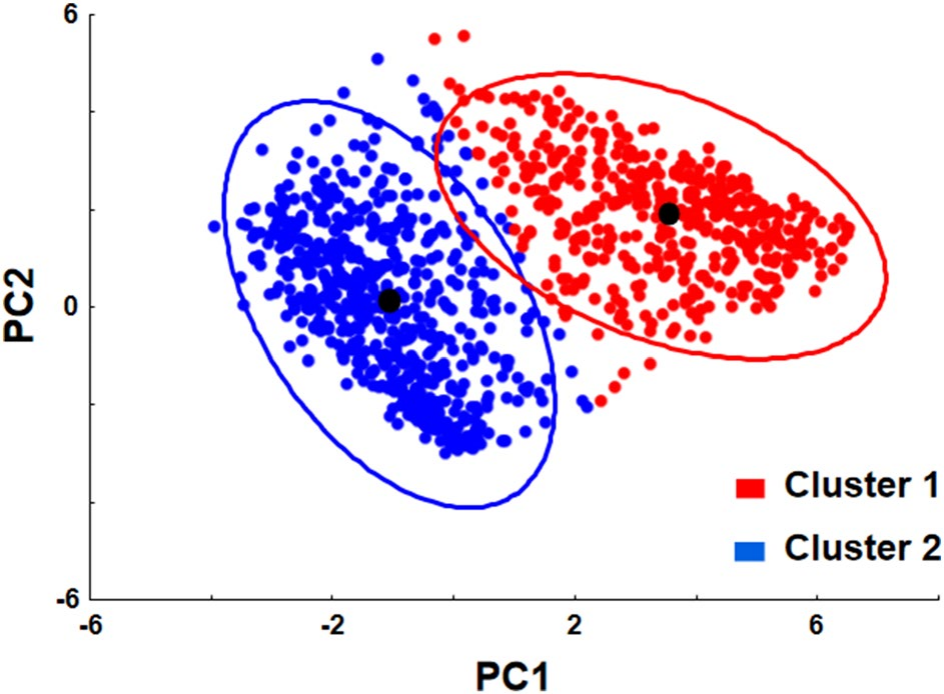
Scatterplot of two principal components from k-means clustering analysis showing the existence of two distinct sperm subpopulations in efferent duct sperm (EDS) after in vitro maturation at 10 s post activation with little overlap. By red and blue colors, the cases of different clusters are marked. The ellipses of 95% confidence for clusters 1 and 2 are presented in red and blue, respectively. Black dots are centroids of corresponding clusters.

Wafer plots at 10 s post-activation time of EDS (Fig. 4) suggested the existence of clearly distinguished sperm subpopulations. Performed cluster analysis allowed the characterization of two subpopulations with very low overlapping of 95% confidence ellipses (Fig. 5) and essential difference in mean values of all kinematic parameters (Table 3).

**Table 3:** Descriptive statistics of kinematic parameters of subpopulations.

Parameter	Cluster N	Average ± SD	Median (interquartile range)
VCL, μm s^−1^	1	164 ± 30	172 (148–187)
	2	58 ± 34	52 (28–80)
VSL, μm s^−1^	1	103 ± 40	103 (71–133)
	2	17 ± 11	14 (9–22)
VAP, μm s^−1^	1	135 ± 30	141 (117–159)
	2	29 ± 17	24 (17–37)
LIN	1	0.63 ± 0.23	0.67 (0.48–0.81)
	2	0.37 ± 0.25	0.32 (0.16–0.59)
STR	1	0.76 ± 0.22	0.83 (0.61–0.93)
	2	0.61 ± 0.27	0.67 (0.39–0.83)
WOB	1	0.82 ± 0.12	0.84 (0.77–0.91)
	2	0.57 ± 0.22	0.56 (0.40–0.75)
ALH, μm	1	4.20 ± 1.35	4.00 (3.20–4.90)
	2	2.92 ± 1.54	2.70 (1.60–3.80)
BCF, Hz	1	7.25 ± 2.51	7.00 (5.90–9.00)
	2	3.70 ± 2.17	3.60 (2.00–5.00)

**Figure 6:**
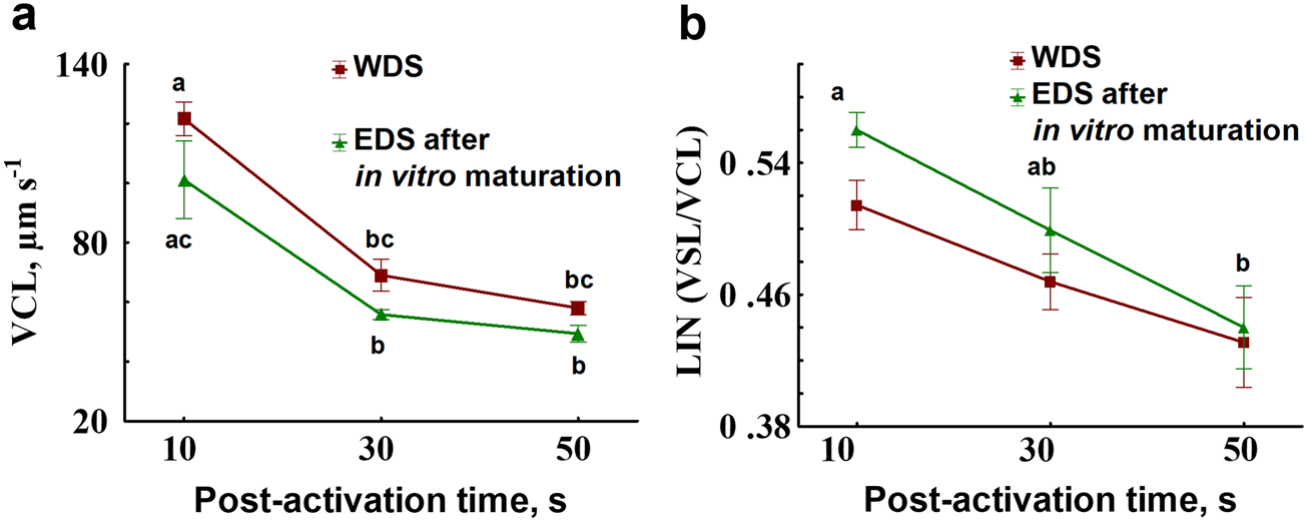
Motility parameters in sperm collected from Wolffian ducts and sperm collected from efferent ducts after in vitro maturation. (**a**)—Curvilinear velocity, µm s^−1^. (**b**)—linearity. EDS—sperm collected from efferent ducts, WDS—sperm collected from Wolffian ducts. Values with different letters are significantly different (Tukey test, *P* < 0.05, for details of the repeated measures ANOVA analysis see Table 4).

The second approach applied for the quantification of differences in motility parameters showed that, after in vitro maturation of EDS, spermatozoa were characterized by VCL and LIN, which were not different from the values detected for WDS (Fig. 6). Results of the mixed model repeated measures ANOVA are presented in Table 4, indicating the significance of factor “post-activation time” for both parameters and significance of factor “sperm type” for VCL only. No significant interaction of factors was found for both parameters.

**Table 4:** Mixed model repeated measures ANOVA statistics obtained for the curvilinear velocity (VCL) and linearity (LIN).

Parameter	Factor	F	P
VCL	Sperm type	5.4593	0.047675
	Post-activation time	19.7208	0.000048
	Sperm type × post-activation time	0.1841	0.833592
LIN	Sperm type	1.503	0.255022
	Post-activation time	5.463	0.015549
	Sperm type × post-activation time	0.177	0.839040

Changes of VCL and LIN in post-activation time were similar for both types of sperm (WDS and EDS after in vitro maturation): VCL significantly decreased at 30 s post activation, while LIN at 50 s (Fig. 6). No significant differences in both parameters were found between WDS and EDS after in vitro maturation at 10, 30, and 50 s post activation.

### Staining of the urogenital system

**Figure 7:**
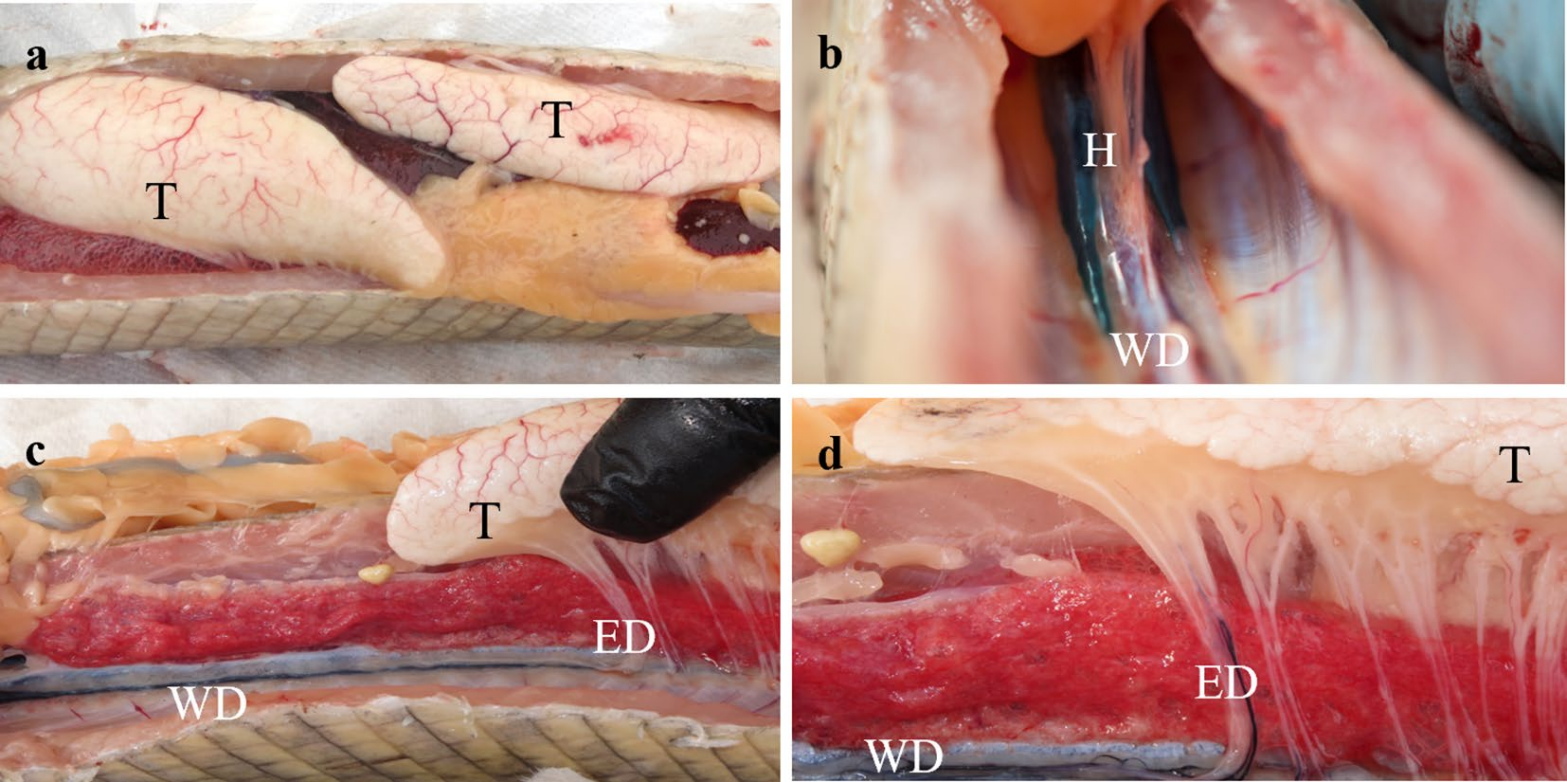
Staining of gar urogenital system. Nigrosin was injected through the urogenital opening. (**a**)—Location of testes (T) in the abdominal cavity. (**b**)—Dye is in horns (H) of the urinary bladder, which is formed by fusion of enlarged posterior portions of Wolffian ducts (WD). (**c**)—Dye is in WD. (**d**)—Dye is entering efferent ducts (ED), connecting WD with testis.

Staining of gar male urogenital system (injection of nigrosine through the urogenital opening) revealed that the dye was slowly progressing through the horns of the urinary bladder and the Wolffian ducts (Fig. 7). Then it easily entered efferent ducts, that means the existence of direct connection between efferent ducts and the Wolffian ducts.

## Discussion

Actinopterygii (except Cladistia) consists of three groups of fishes: evolutionarily more ancient Chondrostei and Holostei and evolutionarily advanced Teleostei. It is well-known that fish speciation was followed by changes in the urogenital system structure. In Teleostei, male reproductive tracts that transport spermatozoa from the testes are fully separated from the excretory system. Thus, spermatozoa and urine are never mixed internally, and any precocious contact of sperm with urine will lead to untimely activation of spermatozoa motility. In ancient groups of Chondrostei and Holostei, the situation is quite different from Teleostei. The excretory and reproductive tracts are not separated.

In sturgeons, sperm ducts enter the kidneys, where sperm mixes with urine before being released into the environment during spawning. This sperm/urine mixing step has been found to be a requisite for post-testicular sperm maturation (PTSM), i.e., getting the capacity to activate motility in the spawning environment^3^. This suggestion was initially based on the detected differences in several sperm parameters. Specifically, it was shown that testicular sperm was characterized by significantly higher sperm concentration and SF osmolality compared to Wolffian duct sperm. Moreover, the ability to initiate motility was acquired by sturgeon testicular spermatozoa only after their transit through the kidneys or after incubation in urine or SF collected from already matured Wolffian duct sperm.

Fish spermatology is quite well developed for teleostean and chondrostean fishes^30^, while sperm biology of holostean fishes is still full of blind spots. Currently, no data related to sperm motility can be found for this fish group. That is why one of the main goals of the present study was to evaluate sperm motility in shortnose gar *Lepisosteus platostomus* (as a representative of Holostei, one of the groups of Actinopterygii) and to explore the occurrence of the PTSM in this fish species occupying, presumably, intermediate position in evolutionary changes in the anatomical association of excretory and reproductive systems. The selection of this holostean fish species for the data presentation was based on our preliminary studies on gar sperm maturation in which we used representatives of three gar species (*L. platostomus*, *L. osseus*, and *L. oculatus*) and did not find any species-specific differences in the structure of their urogenital system and sperm maturation process. This fact as well as the data of Ferrara and Irwin, 2001^31^ who did not find differences in the position of reproductive organs in gars belonging to different genera (*Atractosteus* and *Lepisosteus*) entitle us to apply our conclusions on the whole holostean group.

In the present study, three types of sperm samples were collected: from the testes (TS), from efferent ducts (EDS), and from the Wolffian ducts (WDS). Sperm concentration was significantly changed following sperm transit from the testes to efferent ducts and further to the Wolffian ducts. The TS concentration was more than two and 45 times higher than the values for EDS and WDS, respectively. The EDS concentration exceeded the WDS concentration more than 21 times. The 56-time reduction in sperm concentration of samples collected from the Wolffian ducts compared to those collected from efferent ducts was also documented for sterlet^3^. Such pronounced decrease in gar sperm concentration upon sperm transit from efferent ducts to the Wolffian ducts can be due to the dilution of spermatozoa by urine, which was previously shown for sterlet. An additional confirmation of this possibility can be drawn from the measurement of SF osmolality.

As anticipated, SF osmolality appeared to be significantly lower (more than two times) in WDS compared to EDS samples. The SF osmolality in gar WDS samples (151 ± 33 mOsm/kg) was lower than in freshwater fish species belonging to different taxa but higher in comparison with values for chondrostean species (reviewed by Dzyuba et al.^2^). Similarly to the obtained results, a significant decrease in SF osmolality of WDS compared to that of testicular sperm was reported for sterlet^3^. Based on the decline in sperm concentration and SF osmolality, sperm/urine mixing as a characteristic stage of sturgeon PTSM was suggested in the mentioned previous study. The imperative need of this stage for testicular spermatozoa to become competent to initiate motility in proper conditions was clearly proved, and the method of in vitro maturation of testicular spermatozoa was elaborated^3^.

It was also shown that shortnose gar spermatozoa collected directly from the testes or from efferent ducts could not initiate motility, while spermatozoa collected from the Wolffian ducts became motile in activating medium. The VCL of the latter was around 120 µm s^−1^, which is in a typical range (ca. 110–180 µm s^−1^) found for different freshwater chondrostean and teleostean fish species^3,32–35^. Considering some similarities in the structure of the male urogenital system as well as in the direction of changes in sperm concentration and SF osmolality in representatives of Holostei and Chondrostei, it was reasonable to check the possible post-testicular sperm maturation. For this, a previously developed for sturgeon method of in vitro maturation of testicular spermatozoa through their incubation in SF obtained from naturally mature sperm (collected from the Wolffian ducts) was applied for gar sperm.

After 40 min incubation of TS and EDS in SF obtained from WDS, not more than 5% motile spermatozoa were observed in sperm samples collected directly from the testis. In contrast, the motility percentage in EDS samples rose up to 75%. Performed analysis of sperm kinematic parameters indicated that due to in vitro maturation, shortnose gar spermatozoa acquired motility parameters very close to that of naturally matured (WDS) ones. Both statistical approaches used in the study supported this conclusion. The first approach, applying principal component analysis for the general description of a multivariate CASA dataset followed by cluster analysis, is nowadays considered an appropriate statistical procedure for comprehensive sperm analysis^36^. This approach was used for numerous vertebrate species, with a firm conclusion that subpopulation patterns could have different meanings in different physiological conditions^37^. The sperm maturation process can indeed lead to the appearance of different sperm subpopulations in the ejaculate^38^. That was precisely the case in our study when we found two well-distinguished sperm subpopulations at the initial phase of sperm motility. Considering that TS could not mature in vitro in contrast to EDS, we conclude that the sperm maturation process in gars occurs in several places of reproductive tracts. Efferent ducts, where spermatozoa mix with seminal fluids, probably are the place where the maturation process starts. Mixing EDS with urine leads to final sperm maturation, which is associated with the acquisition of motility potential by spermatozoa.

All the above data mean that, like in sturgeons, the PTSM in gars exists. This maturation can be realized by mixing of testicular spermatozoa with kidney products. At the same time, comparing the results of the present study with data obtained for sterlet^3^ shows a lower dilution rate of gar sperm by urine compared with sterlet. Indeed, sperm concentration and SF osmolality in gar WDS were respectively 21.2 and 2.1 times lower compared to values in EDS, while in sterlet, they were 56 and 7.4 times lower. It can probably be related to the difference in the structure of interconnection of testes and kidneys in these two fish species. In sterlet, testicular spermatozoa go directly to kidneys through efferent ducts that can result in higher dilution rate of sperm by kidney products compared to gars.

Whilst a close connection of testis efferent ducts with kidneys is generally accepted for sturgeon species, not such an unequivocal conclusion can be drawn for gars. The available experimental studies on the structure of gar urogenital organs were mainly undertaken at the end of the XIX–the beginning of the XX century^10,13^. A perfect description of the anatomy, development, and blood supply of gar urogenital system was presented in mentioned studies. Even though some differences in the description can be found in those studies, the authors came to the same conclusion that testicular spermatozoa are transferred by a series of efferent ducts to the kidneys and then to the Wolffian ducts, which means that there is no direct contact between efferent ducts and the Wolffian ducts found. If this is the case, gar male urogenital system structure resembles that in sturgeons described by Wrobel and Jouma^39^. They state that Acipenser “spermatozoa have to travel through Bowman's capsules and tubules of the nephrons involved, then through the urinary collecting ducts, the wolffian duct and finally sinus urogenitalis” that means that before entering the Wolffian duct sperm is carried by fully functional renal units simultaneously with the excretory products. Notwithstanding the foregoing, an alternative statement can be found in modern literature. According to Lahnsteiner and Patzner^12^, in the Holostei, the testes form a secondary sperm duct that joins with the posterior portion of the Wolffian duct, meaning that excretory and seminal ducts are almost separated.

**Figure 8:**
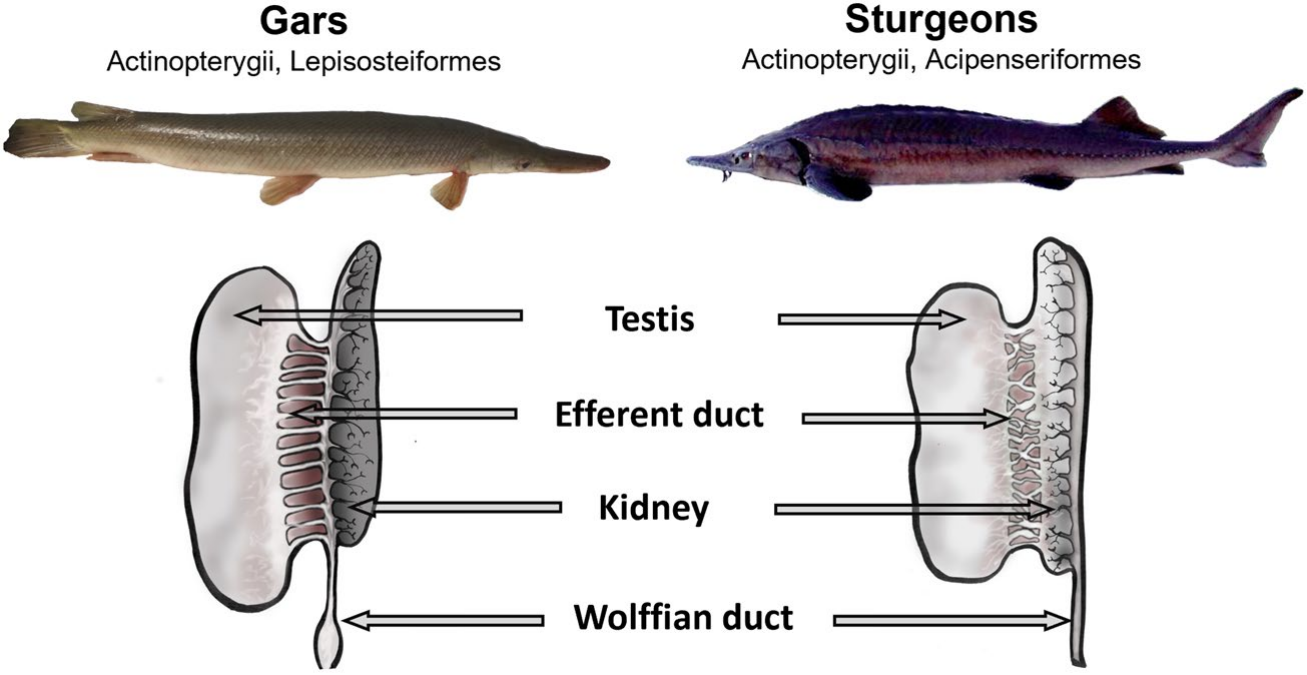
Schematic presentation of differences in the urogenital structure of gars and sturgeons.

The injection of nigrosin through the urogenital opening in the present study showed that the dye was slowly progressing through the horns of the urinary bladder formed by the fusion of enlarged distal portions of the Wolffian ducts, then the Wolffian ducts and, after that, easily entered the efferent ducts within the mesorchium. Such easy dye entering the efferent ducts in gar contradicts our previous observations made during similar staining of sterlet urogenital system (not published personal data). In sterlet, no entry of the dye into the efferent ducts happened. This difference between gar and sterlet means that in Lepisosteidae, in contrast to Acipenseridae, efferent ducts transporting spermatozoa from the testes enter directly the Wolffian ducts, not kidneys (Fig. 8). At the same time, lower sperm concentration and SF osmolality in semen collected from the Wolffian ducts strongly imply the mixing of testicular spermatozoa with urine. How exactly spermatozoa can be mixed with kidney products still needs further clarification.

## Conclusions

For the first time, sperm motility and post-testicular sperm maturation were studied in the shortnose gar *Lepisosteus platostomus*, a representative of Holostei. The presented data clearly show that post-testicular sperm maturation occurs in gars. The decrease in seminal fluid osmolality during post-testicular sperm maturation makes this process similar to that previously described in sturgeons. In sturgeons, this decrease results from sperm mixture with urine due to direct contact between the testes and kidneys. At the same time, the absence of such contact in Holostei makes them similar to evolutionarily advanced Teleostei, in which urine can not be involved in sperm maturation. So, we consider Holostei as a fish taxon, which occupies an intermediate evolutionary position between Teleostei and Chondrostei in the anatomical arrangement of excretory and reproductive systems. For future elucidation of sperm maturation processes in holostean fishes, the study should be continued to describe more precisely the contact of Wolffian ducts with kidneys (including application of modern histological techniques) to understand if post-testicular sperm maturation requires contact with urine. To open new perspectives in understanding the evolution of urogenital system anatomy and sperm maturation strategies in vertebrates, the changes in spermatozoa functionality during their transit through efferent ducts (preparation for maturation) should also be addressed.

## Materials and methods

### Ethics approval

We confirm that the current study was performed according to ARRIVE guidelines and all experiments were conducted in compliance with the principles of the local ethics committees of the University of Oklahoma, OK, USA, and the University of South Bohemia in Ceske Budejovice, Czech Republic. The study protocol was approved by the University of Oklahoma, Institutional Animal Care and Use Committee.

### Fish

The study was done using shortnose gars *Lepisosteus platostomus* (Holostei, Lepisosteiformes, Lepisosteidae). Nine mature males of *L. platostomus* (weight 1.1 ± 0.2 kg; total length 63 ± 3 cm) were transferred from outdoor research ponds (water temperature 12 °C) of the Aquatic Research Facility, Biology Department, the University of Oklahoma, to the laboratory fish tanks, where the water temperature was increased to 20 °C within two days. Fish were held in this condition for two more days, after which fish were injected with calibrated carp pituitary extract (CCPE, Israel) at 0.3 mL kg^−1^ (dose: 6 mg of extract per kg of body weight). Sperm was collected 48 h after hormonal treatment.

### Collection and processing of sperm samples

For sperm collection, fish were anesthetized on crashed ice and then decapitated. After dissection, three types of sperm samples were collected: sperm from the testes (TS), from efferent ducts (EDS), and from Wolffian ducts (WDS). Sperm concentration in all collected samples was measured using a Burker cell hemocytometer (Meopta, Prerov, Czech Republic) and Olympus BX50 phase contrast microscope (200× magnification; Olympus, Tokyo, Japan). To get seminal fluids (SF), sperm samples were centrifuged at 4 °C (3,000 g, 30 min). The osmolality of SF was measured using a freezing point osmometer Osmomat 030 (Gonotec GmbH, Berlin, Germany). Three replicates for each SF were measured, then mean values for individual SFs were averaged.

For in vitro maturation of testicular sperm, the recently developed method of in vitro sterlet testicular sperm maturation^3^ was used. It involves testicular sperm incubation in SF derived from mature Wolffian duct sperm. In the present study, TS and EDS were incubated in SF from WDS at a dilution rate of 1 volume of TS/EDS to 9 volumes of SF for 40 min. Incubation was done at room temperature.

### Experimental dyeing of urogenital ducts

After fish dissection, dyeing of male gar urogenital ducts was done using nigrosin (10% w/w in water). The dye was gradually injected through the urogenital papilla, and its propagation toward the testis was documented by photography.

### Sperm motility video recording and data acquisition

Motility parameters of all sperm samples (WDS, TS, and EDS before and after in vitro maturation) were evaluated after sperm motility initiation by dilution of sperm in activating solution consisting of 10 mM Tris–HCl, pH 8.2, and 0.25% Pluronic F-127. Sperm dilution was made on the surface of the microscopy slide by injection needle (dilution rate approximately 1:1000) to get 20–50 spermatozoa visible in the field recorded by the video camera. Microscope focusing was made on the bottom of the drop (water/glass interface). Video microscopy was performed using CCD video camera (SONY, SSCDC50AP) mounted on a dark-field contrast microscope (Olympus BX50, Tokyo, Japan). Video records were converted into 25 fps, grayscale video avi files and analyzed using the integrated system for semen analysis (ISAS, Proiser, Spain). Percent of motile cells (motility percent, %), kinematic parameters as VCL (curvilinear velocity, μm s^−1^), VAP (average path velocity, μm s^−1^), VSL (straight-line velocity, μm s^−1^), LIN (linearity of the path, VSL/VCL), WOB (oscillation of the track, VAP/VCL), STR (trajectory straightness, VSL/VAP), BCF (beat-cross frequency, Hz), ALH (amplitude of lateral movement of the sperm head, μm) were evaluated. As the duration of motility in all samples did not exceed 60 s, sperm motility analysis was performed at 10, 30, and 50 s post activation to characterize sperm motility at the initial, middle, and end phases of movement. The limit for distinguishing motile spermatozoa was set at VCL = 10 µm s^−1^.

### Statistical analysis

One-way ANOVA followed by posthoc analysis using the Tukey test was performed to compare motility percent, SF osmolality, and sperm concentration in different sperm types (WDS, TS, and EDS before and after in vitro maturation). Values were assessed by Shapiro–Wilk and Levene tests to check the normality and homogeneity of variances in these experimental groups. No assumption of normality and homogeneity of variances was violated for SF osmolality and motility percent, while values of sperm concentration were Box-Cox transformed for ANOVA analysis.

Kinematic parameters of spermatozoa obtained by CASA for all sperm samples used in the study were analyzed using two approaches. The first approach was performed to visualize the general structure of CASA data by multivariate exploratory techniques using principal component (PC) analysis. In this approach, the entire dataset of sperm motility parameters of each tracked spermatozoon was normalized [subtracting a sample mean from each value and dividing it by sample standard deviation (SD)]. Normalized data were used for calculating motility parameters loadings to three PCs with the highest eigenvalues. These loadings were used to calculate the PC coordinates of each spermatozoon, followed by the presentation of these data as separate Wafer plots (3D scatterplot for WDS and EDS at three post-activation times) and with the representation of data centroids (the dot with averaged PC1–PC3 coordinates). When visually clearly distinguished sperm subpopulations were visualized on Wafer plots (e.g., at 10 s post activation in EDS), the data of two PCs with eigenvalues higher than one (according to Kaiser criterium) were subjected to k-means clustering. The obtained coordinates of each event (spermatozoon) for each cluster were used to contrast cluster location by plotting 95% confidence (prediction) ellipses and to calculate the coordinates of the cluster centroids. In the second approach, oriented on quantifying differences in motility parameters, kinematic parameters of spermatozoa obtained by CASA for all sperm samples used in the study were initially analyzed using Spearman's rank correlation coefficient. For simplifying data presentation, only parameters with a low correlation coefficient (r < 0.1) were selected as descriptors of sperm movement. These parameters were VCL (curvilinear velocity) and LIN (linearity). The obtained for individual spermatozoon VCL and LIN data were averaged for each male. These averaged data were used for further analysis. After the check of the normality and homogeneity of variances of these data by Shapiro–Wilk and Levene tests, mixed model repeated measures ANOVA was performed to identify the differences among groups, considering sperm type (WDS, EDS) as a categorical factor, post-activation time (10, 30, 50 s) as within-subject (repeated measures) factor, and VCL and LIN as dependent factors. As no significant interaction between factors was detected, further posthoc analysis was performed according to Wei et al.^40^: (1) if there was a main effect for only one factor, multiple comparisons among the treatment means for this factor were performed to identify which specific means differ; (2) if there were main effects for both factors, comparisons of group means were performed.

All statistical analyses were performed using Statistica (data analysis software system, version 13, TIBCO Software Inc. (2018), http://tibco.com). Statistical hypotheses in the applied tests were rejected at *P* < 0.05. Data are presented as the arithmetic mean and standard error in figures and the arithmetic mean and SD in the text.

## Data Availability

The data presented in this study are available on request from the corresponding author.
